# H-NS uses an autoinhibitory conformational switch for environment-controlled gene silencing

**DOI:** 10.1093/nar/gky1299

**Published:** 2018-12-28

**Authors:** Umar F Shahul Hameed, Chenyi Liao, Anand K Radhakrishnan, Franceline Huser, Safia S Aljedani, Xiaochuan Zhao, Afaque A Momin, Fernando A Melo, Xianrong Guo, Claire Brooks, Yu Li, Xuefeng Cui, Xin Gao, John E Ladbury, Łukasz Jaremko, Mariusz Jaremko, Jianing Li, Stefan T Arold

**Affiliations:** 1 King Abdullah University of Science and Technology (KAUST), Computational Bioscience Research Center (CBRC), Division of Biological and Environmental Sciences and Engineering (BESE), Thuwal, 23955-6900,Saudi Arabia; 2 Department of Chemistry, The University of Vermont, Burlington, VT 05405, USA; 3 Department of Physics (IBILCE), São Paulo State University, São José do Rio Preto, São Paulo, Brazil; 4 King Abdullah University of Science and Technology (KAUST), Imaging and Characterization Core Lab, Thuwal, 23955-6900, Saudi Arabia; 5 King Abdullah University of Science and Technology (KAUST), Computational Bioscience Research Center (CBRC), Computer, Electrical and Mathematical Sciences and Engineering Division (BESE), Thuwal, 23955-6900, Saudi Arabia; 6 School of Molecular and Cellular Biology, University of Leeds, Leeds, UK; 7 King Abdullah University of Science and Technology (KAUST), Division of Biological and Environmental Sciences and Engineering, Thuwal, 23955-6900, Saudi Arabia

## Abstract

As an environment-dependent pleiotropic gene regulator in Gram-negative bacteria, the H-NS protein is crucial for adaptation and toxicity control of human pathogens such as *Salmonella, Vibrio cholerae* or enterohaemorrhagic *Escherichia coli*. Changes in temperature affect the capacity of H-NS to form multimers that condense DNA and restrict gene expression. However, the molecular mechanism through which H-NS senses temperature and other physiochemical parameters remains unclear and controversial. Combining structural, biophysical and computational analyses, we show that human body temperature promotes unfolding of the central dimerization domain, breaking up H-NS multimers. This unfolding event enables an autoinhibitory compact H-NS conformation that blocks DNA binding. Our integrative approach provides the molecular basis for H-NS–mediated environment-sensing and may open new avenues for the control of pathogenic multi-drug resistant bacteria.

## INTRODUCTION

By controlling the expression of >200 genes in an environment-dependent manner, the histone-like nucleoid-structuring (H-NS) protein contributes crucially to the fitness of enterobacteria, including pathogenic and multidrug resistant strains (1–4). Under non-permissive metabolic and environmental conditions, H-NS represses gene expression by coating and/or condensing (bridging) DNA strands (5–7). Repression is thought to result from H-NS molecules (i) blocking access of the RNA polymerase from the promoter, or (ii) trapping the RNA polymerase, or (iii) interfering with RNA polymerase progression by intragenic binding (8–12). By preferentially binding to AT-rich DNA sequences, H-NS controls in particular foreign DNA (‘xenogeneic silencing’) and pathogenic islands (13–18). H-NS releases DNA to enable gene expression, depending on the growth phase, but also in response to changes in environmental factors, such as temperature, osmolarity and pH (4,19–21). This mechanism is thought to allow bacteria to sense their presence within a warm-blooded host and adapt the bacteria's response accordingly. For pathogenic enterobacteria (including *Salmonella, Vibrio cholerae*, and pathogenic *Escherichia coli*) this mechanism also permits the controlled expression of toxicity islands (14,22,23). Therefore, a detailed understanding of environment-sensing by H-NS may also open ways to lower the toxicity and fitness of pathogens. The current consensus is that an increase in temperature (from ambient to the 37°C inside of the animal host) disrupts the self-association of H-NS, which weakens its grip on DNA, and hence its capacity to compact DNA and to repress gene expression (24). However, the molecular basis for environment-sensing by H-NS is unclear and remains controversial (4,25–29).

**Figure 1.**
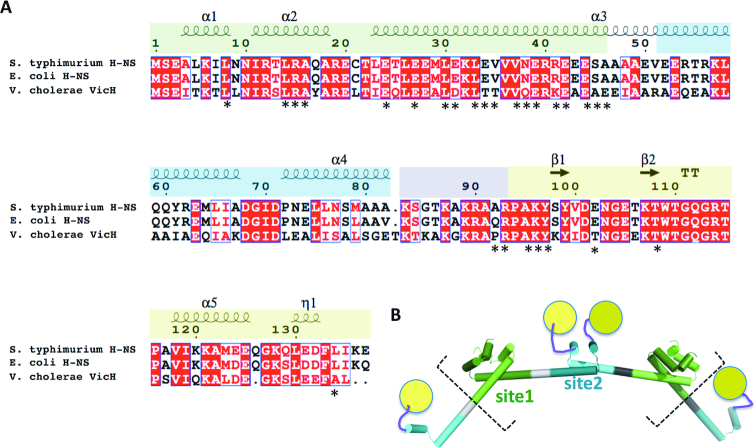
Domain organization of H-NS. (**A**) Sequence alignment showing structural elements (α-helices, β-strands; η represents a 3_10_ helix). Protein domains (determined from experimental structures) are green: N-terminal dimerization domain (site1); blue: central dimerization domain (site2), purple: positively charged linker; yellow: C-terminal DNA binding domain (DNAbd). Positions of *S. typhimurium* residues showing chemical shift changes in presence of H-NS_84–137_ or H-NS_2–57_ are indicated by an asterisk. (**B**) Schematic structural model for multimerization through site1 (light and dark green) and site2 (light and dark cyan) extracted from the H-NS superhelix conformation (see (35) and PDB 3NR7). The charged linker (purple) and DNAbd (yellow) are not included in the crystallographic model. Dashed square brackets outline the construct used in MD simulations.

H-NS is composed of an N-terminal dimerization domain (site1, residues 1–44), a second, central dimerization domain (site2, residues 52–82) and a C-terminal domain involved in DNA binding (residues 93–137; DNAbd. Figure 1). To associate with DNA, H-NS uses mainly its C-terminal region, including a charged linker region (residues 84–93) and the DNAbd (30–32). The N-terminal region also contributes to DNA interactions, however in an incompletely understood manner (25). At least two modes of DNA-binding appear possible; H-NS can multimerize along DNA strands, thus stabilizing (‘stiffening’) supercoiled DNA topology; H-NS can also bridge adjacent DNA strands, linking distant DNA regions or stabilizing DNA-loops (4–6,9,27,33–38). DNA interactions of each mode might differ, because H-NS mutants can selectively block DNA stiffening without disrupting DNA bridging (8), and changes in the concentration of divalent cations can tip the balance from one mode to the other (33,39–41), although quantification of these effects is extremely difficult. The molecular details for both DNA interaction modes, and their relative functional importance are still insufficiently understood (38).

**Figure 2.**
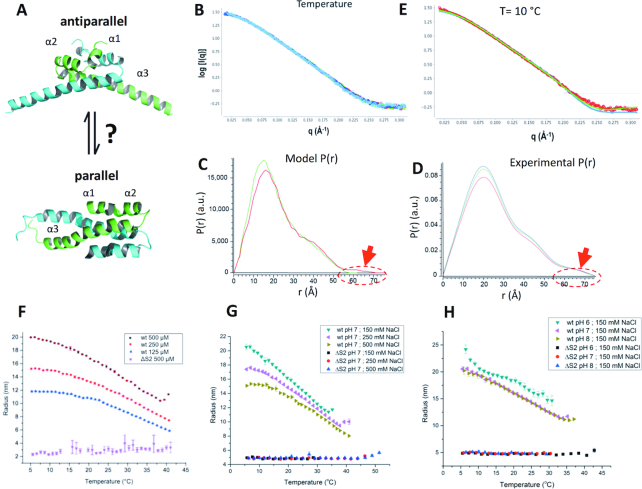
Environmental influence on the structure of S. typhimurium H-NS. (**A**) Structural models for site1 (residues 1–57 are shown). *Top*: antiparallel model, as observed in *E. coli* H-NS_1–46_ (1NI8, NMR), *S. typhimurium* H-NS_1–83,C21S_ (3NR7, X-ray), *S. typhimurium* H-NS_1–46_ (4ICG, X-ray) and *V. cholerae* VicH_1–48_ (1OV9, X-ray). *bottom*: parallel model, as determined for *S. typhimurium* H-NS_1–57,C21S_ (1LR1, NMR). The two chains of each dimer are coloured green and cyan. (**B**) Superimposed are SAXS data collected on *S. typhimurium* H-NS_2–57,C21S_ at 10°C (light blue), 20°C (dark blue), 30°C (green) and 42°C (red). Data were collected in 150 mM NaCl, 1 mM EDTA, 20 mM Tris pH 7.5. (**C**) Pair distance distributions *P*(*r*) calculated for the parallel (green) and antiparallel (red) models for H-NS_1–57,C21S_. The encircled area and red arrow highlight a main difference between the both site1 models. (**D**) *P(r)* for H-NS_2–57,C21S_, derived from SAXS data shown in (D); 20°C (blue), 30°C (green) and 42°C (red). (**E**) Fit of calculated SAXS pattern (green) to experimental data (red dots) collected at 10°C. Green: antiparallel model; χ^2^ = 2.22. Blue: parallel model; χ^2^ = 7.56. Fits were calculated by FoXS (57). (F–H) DLS measurements of *R_H_* as a function of temperature. (**F**) Temperature and concentration dependence on multimerization of H-NS without fused mCherry. Wt: full-length H-NS; ΔS2: H-NSΔs2. (**G**) Influence of salt on multimerization of ^mCh^H-NS (wt) and ^mCh^H-NSΔs2 (ΔS2). (**H**) as (G) but with variations in pH. For other salt and pH scans, please see Supplementary Figure S3. Data in (F–H) are means ± S.D., *n* = 3.

Our previous NMR and crystallographic analyses of H-NS from *E. coli* and *Salmonella typhimurium* revealed that site1 forms a 3-helical dimerization domain (Figures 1, 2A) (25,29). Through structural analysis of H-NS_1–83_ we further showed that H-NS site1 dimers can associate into a superhelical structure in a head-to-head/tail-to-tail manner through connections established by site2, which forms a helix-loop-helix dimerization element (Figure 1B) (35). This analysis provided a mechanistic basis for how H-NS multimerizes along DNA, resulting in stiffening of supercoiled DNA. *In vitro*, the multimeric structure formed by H-NS_1–83_ disassembles into dimer-sized particles upon increasing the temperature above 37°C, suggesting that H-NS_1–83_ contains the site for temperature sensing (35).

Following the publication of two different NMR structures for the H-NS site1 (a parallel (29) and an antiparallel fold (25)), it was proposed that environment sensing involves a conformational rearrangement of site1 between the parallel and antiparallel folds (4) (Figure 2A). Observations from dynamic force spectroscopy suggested that the parallel H-NS structure prevailed under the conditions used in these experiments (27), whereas all crystal structures showed site1 only in the antiparallel fold (26,35,42), despite having been determined under different experimental conditions. A subsequent computational analysis, based on 40-ns molecular dynamics (MD) simulations, found that the parallel fold of site1 was only stable at high salt concentrations (500 mM NaCl) and in presence of a C21S mutation, whereas the antiparallel fold was stable for all tested conditions and sequences (28). Indeed, the NMR structure of the parallel fold was determined for the *S. typhimurium* H-NS_1–57,C21S_ construct at 300 mM NaCl, whereas the H-NS_1–83,C21S_ construct crystallized in the antiparallel fold in the absence of inorganic salt (29,35). Although these MD simulations were compatible with the existence of a parallel conformation of H-NS site1 under specific conditions, a parallel-to-antiparallel transition in response to physicochemical changes has neither been observed in simulations nor in experiments.

An alternative model has recently been proposed based on MD simulations (41). In some of these simulations, a ‘buckling’ of the central region of helix α3 (residues 42–50) allowed forming compact ‘closed’ H-NS conformations where the C-terminal domain contacts the N-terminal dimerization domain. The DNA-binding regions of the C-terminal domain were buried in these interactions, suggesting a conformational switch that inactivates DNA-binding by H-NS (41). However, direct experimental evidence for the intramolecular interactions between the N-terminal and C-terminal H-NS regions and for helix 3 buckling have not been provided.

Here, we combine structural, biophysical and computational methods to investigate the molecular basis for environment-sensing of H-NS. Our results provide compelling evidence against a parallel-to-antiparallel switch mechanism of site1. Instead, they reveal an autoinhibitory mechanism where heat-induced unfolding of site2 allows interactions between the N-terminal and C-terminal domains of H-NS. The resulting ‘closed’ conformation is incompatible with DNA binding, explaining release of gene repression by H-NS at human body temperatures.

## MATERIALS AND METHODS

### Protein production

*Salmonella typhimurium* full length H-NS_C21S_, H-NS_1–83,C21S_ and H-NS_2–57,C21S_ were cloned and produced as described previously (52). H-NS_91–137_ was cloned into a pLICb.HM ampicillin resistant plasmid as a 6His-Maltose fusion protein. *Escherichia coli* production of H-NS_91–137_ was as reported for H-NS_2–57,C21S_, except that 50 μg/ml ampicillin were used, and that the flasks were cooled to 18°C for 16 h following induction with 1 mM isopropyl β-d-1-thiogalactopyranoside (IPTG). Frozen cell pellets were thawed on ice in a buffer containing DNase, 1 mM PMSF, 2 mM βME, 20 mM potassium phosphate, 500 mM NaCl and 5 mM imidazole at pH 7.5. Resuspended bacteria were lysed on ice by sonication, followed by microfluidization, and centrifuged in the same manner as H-NS_2–57._ The supernatant was loaded onto a preequilibrated (in 20 mM potassium phosphate, 500 mM NaCl, 5 mM imidazole at pH 7.5) His-trap FF 5 ml global NiNTA column (G.E. Healthcare). The protein was washed with 500 mM NaCl, 20 mM potassium phosphate, 5 mM imidazole at pH 7.5 for 15 column volumes, before elution via a linear gradient of 10 column volumes from 5 mM imidazole to 200 mM imidazole, and then a linear gradient of 10 column volumes from 200 mM imidazole to 1 M imidazole with 20 mM potassium phosphate and 500 mM NaCl. The fractions containing the protein were pooled, and TEV protease at 1ml/100 ODU was added. Immediately, the protein was dialyzed at 4°C against a 20 mM potassium phosphate, 500 mM NaCl, 2 mM βME solution. Next, the cleaved His-MBP tags were removed by passing the protein solution again through the NiNTA column using the same buffers as before and collecting the flow through. The flow through was concentrated to 5 ml, and loaded onto a 120 ml Superdex 75 column (GE Healthcare) preequilibrated with 300 mM NaCl, 20mM potassium phosphate, 2 mM beta-mercaptoethanol and 1 mM EDTA at pH 7.0. The fractions with the protein were pooled and concentrated as required for biophysical analysis.

For MST, NMR and ITC analysis, the gene encoding the N-terminus (2–57) of H-NS was cloned into the pET-M vector [modified pET-32a (+) vector, Novagen] with an N-terminal His_6_-tag and the H-NS_2–57_, H-NS_91–137_ and H-NS_84–137_ constructs were cloned into a pGEX 6P-1 vector with N-terminal GST tag. The resulting plasmid of N-terminus was transformed into BL21 (DE3) cells. The transformed cells were incubated at 37°C until OD_600_ of 0.6 and induced with 0.3 mM IPTG at 20°C for 16 h. Harvested cells were resuspended in lysis buffer: 50 mM Tris–HCl (pH 7.0), 200 mM NaCl and 1 mM dithiothreitol (DTT), sonicated on ice for 10 min and centrifuged at 25 000 g for 30 min. The supernatant was incubated with Ni-NTA beads for an hour at 4°C and then washed twice with wash buffer: 50 mM Tris–HCl (pH 7.0), 200 mM NaCl, 1 mM DTT and 5 mM imidazole. The protein was eluted with 250 mM of imidazole in the lysis buffer. The eluted protein was loaded onto a Superdex75 size exclusion column (GE Healthcare) equilibrated with buffer containing 20 mM sodium phosphate (pH 6.5), 50 mM NaCl and 3 mM DTT. The protein was concentrated to 30 mg/ml. The plasmids of H-NS_2–57_, H-NS_91–137_ and H-NS_84–137_ were transformed into BL21 cells. For ^15^N labeling of H-NS_2–57_, H-NS_91–137_ and H-NS_84–137_, the cells were grown in M9 minimal media supplemented with ^15^N ammonium chloride (Cambridge Isotopes Laboratories) until OD_600_ of 0.6 at 37°C and induced with 0.15 mM IPTG at 16°C for 16 h. The above protein purification protocol was adapted for ^15^N labeled H-NS_2–57_, H-NS_91–137_ and H-NS_84–137_ with exception of GST tag was cleaved overnight at 4°C using 3C Protease. Eluted protein was loaded onto a Superdex75 size exclusion column (GE Healthcare) equilibrated with buffer containing 20 mM sodium phosphate (pH 6.5), 50 mM NaCl and 3 mM DTT.

### Design of a short 101 bp DNA sequences for H-NS binding studies

The 101 bp DNA sequence was designed using Virtual Footprint (http://www.prodoric.de/vfp/) based on (53). The whole *E. coli* K12 chromosome was searched for the best H-NS binding sites. Next we identified a 100 bp region containing at least three sites with a good score. Region 1051777 to 1052016 on the *E. coli* K12 chromosome was then selected based on Virtual Footprint results.

### Thermal proteolysis

1 mg/ml thermolysin solution was prepared using buffer provided by Hampton Research. The proteolysis assay buffer contained 20 mM HEPES buffer at pH 7.5 and 100 mM NaCl and 10 mM CaCl_2_ for purified proteins. Digestion of 2 mg/ml protein was performed in a T100 thermal cycler (Biorad) with addition of 1 μg of thermolysin to 100 μg of proteins for one hour at 25 and 37°C respectively (54). The reaction was quenched with addition of EDTA at final concentration of 50 mM. Equal protein volumes were analyzed on 20% SDS PAGE using Coomassie stain. Pictures were taken using GelDoc (Biorad).

### Thermal stability of proteins from rates of oxidation (SPROX) using MALDI-TOF

Three aliquots of 10 μM protein samples were kept in 20 mM HEPES, pH 7.5, 100 mM NaCl. For the thermal SPROX analyses, 9 μl aliquots of the protein samples were equilibrated for 20 min at temperatures of 25 and 37°C. The oxidation reaction was initiated at each temperature with the addition of 2 μl of 1 M H_2_O_2_ to give a final concentration of 200 mM H_2_O_2_. After incubation for 1 h, 300 mM methionine solution was added, and the samples were analyzed by MALDI-TOF mass spectrometry to determine the extent of protein oxidation (55).

### Dynamic light scattering

For DLS measurements, H-NS from *S. typhimurium* was expressed as N-terminal mCherry fusion proteins with an N-term His tag in *E. coli* BL21 using the expression vector pET28b. The linker sequence SAGGSASGASG was inserted between mCherry and H-NS proteins to avoid steric clashes in the dimer. Bacteria were grown in LB medium, induced with 1mM IPTG at 25°C overnight. Cells were harvested and resuspended in lysis buffer (50 mM Tris pH8, 500 mM NaCl, 10 mM Imidazole with addition of lysozyme, DNase I and 1% triton X-100) and lysed by mild sonication. Proteins and bacterial membranes were separated by centrifugation (30 min, at 15 000 × *g*) and the supernatant was applied to Ni-NTA beads (Qiagen) for 2 h. The column was washed thoroughly with 50 mM Tris pH8, 500 mM NaCl, 10 mM Imidazole and protein was then eluted with 50 mM Tris pH 8, 500 mM NaCl, 400 mM Imidazole, 1 mM DTT. After dialysis in 50 mM HEPES pH 7.4, 300 mM NaCl, 0.5 mM TCEP, eluted protein was further purified by ion-exchange chromatography using either MonoQ or MonoS column (GE) in the same buffer. Protein multimerization was observed in combination of different salt (150, 250 and 500 mM NaCl) and pH (6, 7 and 8) conditions. For this, 100 mM MES, MOPS and HEPES buffers were used, with proteins at concentrations ranging from 125 to 500 μM, in a final volume of 100 μL. Dynamic light scattering measurements were performed in 96-well plates (Greiner) using a DynaPro plate reader-II (Wyatt Technologies). A triplicate of three wells was measured for every sample with 5 acquisitions of 5 s for every well. The machine was cooled with gaseous nitrogen, with a starting temperature of 5°C, followed by an increase to 60°C at a ramp rate set so that each well is measured every 1°C. Data were analysed with DYNAMICS software (Wyatt Technologies) as Temperature Dependence and exported for further fitting on Origin software using a Logistic Fit. The presented results are mean values with standard error mean determined from the triplicate sample.

### SAXS analysis

H-NS_FL_ and H-NS_2–57_ Data were recorded at the ALS, Berkeley, CA, beamline 12.3.1. using a wavelength of 1 Å. Buffer conditions are stated in the figure legends. Measurement temperatures were 10°C unless stated otherwise. Scattering data were recorded on protein concentration series between 4 and 1 mg/ml, and on the exactly matching buffers (taken from the final dialysate of two 500 ml, 3 h protein dialyses). Buffer scattering was recorded before and after the protein samples, controlled for consistency, and subtracted from the scattering of the protein samples. SAXS data on H-NSΔs2 were recorded at the SWING beamline (SOLEIL, Saint-Aubin, France) with λ 1.03 Å. The distance of the sample to the detector was 1.8 m, resulting in the momentum transfer range of 0.01 Å^−1^ < *q* < 0.5 Å^−1^. Buffer data were calculated from the buffer (20 mM Tris pH 7.5, 50 mM/750 mM NaCl, 3 mM DTT) eluted before proteins, and subtracted from the protein data using SWING’s on-site software. Data were analysed using PRIMUS, BUNCH, DAMMIN, DAMMIF and DAMAVER of the ATSAS software package (56), ScÅtter (http://www.bioisis.net) and FOXS (57). The *P*(*r*) plots for the 3D structural models were calculated with moleman2.

### Nuclear magnetic resonance

300 μl of ^15^N-labeled H-NS_91–137_ and H-NS_84–137_ at a concentration of 500 μM was used for ^1^H–^15^N HSQC measurements, with 2 μl of 25 mM 2,2-dimethyl-2-silapentane-5-sulfonate (DSS) sodium salt as an internal chemical shift reference for ^1^H at 0 ppm. For ^1^H–^15^N HSQC titration studies, unlabeled H-NS_2–57_ was added to ^15^N-labeled H-NS_91–137_ and H-NS_84–137_ until the ratio of 0.5:1, 1:1 and 2:1 respectively. The above protocol was adapted for titration of unlabeled H-NS_84–137_ to ^15^N-labeled H-NS_2–57_ until the ratio of 4:1. Changes in chemical shifts for ^1^H and ^15^N were measured in ppm ($\delta H$ and $\delta {\rm{N}}$) where the ^15^N shift changes were multiplied by a scaling factor $\alpha = 0.14$, and then the summed Euclidean distance moved were calculated following this equation: ${\rm{d}} = \,|\delta H| + \alpha |\delta {\rm{N}}|$ (58). The ^1^H–^15^N HSQC titration experiments were carried out at a temperature of 25°C using a Bruker Avance III 950 MHz NMR spectrometer, equipped with a triple resonance inverse TCI CryoProbe. Spectra were acquired with 2048 (^1^H) × 200–256 (^15^N) complex points, a spectral width of 16 ppm for ^1^H and 40 ppm for ^15^N and averaged for 50 scans.

### Calculation of the hydrodynamic radius of H-NS species

The 3D structures of different forms of H-NS namely, ^mChe^H-NS dimer, ^mChe^H-NSΔs2 dimer and ^mChe^H-NS multimers were built using PyMol and Swiss Model (59) based on PDB entries 3NR7, 2L93 and 5FHV WinHYDROPRO 1.0 (GUI) was used to calculate the hydrodynamic radius using the translational diffusion coefficient (Dt) for each structure modeled (60). HYDROPRO uses the coordinates from the PDB file at the atomic as well as residue level. The program also needs simple supplementary data such as temperature, molecular weight and the type of calculation. The type of calculation was set to the atomic level with the AER value of 2.9 Å, as recommended by the developers when using atomic level shell calculation. The solution viscosity was set to 0.01, adapted for non-viscous aqueous buffers (60). The protein molecular weight was calculated using the protein sequence by ProtParam (61). Based on this input, WinHydroPro produced the value for the radius of gyration (*R*_g_), but not the hydrodynamic radius. We calculated the hydrodynamic radius with the help of translational diffusion coefficient provided by the WinHydroPro output. The translational diffusion coefficient (Dt) is expressed as Dt = *kT/f* (where f is the friction coefficient, *k* is the Boltzmann constant and *T* is temperature (in K). The formula to calculate the hydrodynamic radius using the diffusion coefficient can be expressed by *R*_H_ = *kT*/6*π*η*Dt (where *π* = 3.14, *k* = Boltzman constant (1.38064852 × 10^−23^ m^2^ kg s^−2^ K^−1^), temperature = 293 K and η = 0.01 Pa s (viscosity)) (3). The values of *R*_H_ were calculated for all the models using the above equation.

### Atomistic MD simulations

#### Model preparation

We built the H-NS homology models from template PDBIDs: 3NR7 (H-NS_1–83,C21S_) and 2L93 (DNAbd_91–137_) using Prime (Schrödinger, Inc.). Each dimer model contains two full-length monomers dimerized at site2 in the middle, and the N-term of each monomer forms an antiparallel site1 dimer with another truncated site1 monomer (residues 2–49); the C-terminal DNAbd was connected to site2 by a stretched-out loop region. All simulations were performed with the CHARMM36-cmap force field (62). We prepared the solvated systems with a previously described protocol (63) and performed MD simulations using VMD (64), NAMD (65) or ANTON (66). Each system contains a protein model, ∼33 400 TIP3P water molecules, counter ions, and 150 or 500 mM NaCl, totalling ∼106 300 atoms in a periodic box ∼133 × 98 × 98 Å^3^.

#### Simulation setup

Each system went through minimization, 250 ps equilibration, and 200 ns MD production procedure in the NPT ensemble (20 or 42°C, 1 bar) with a time step of 2 fs. Langevin dynamics (67) with low damping coefficient of 1 ps^−1^ was used for temperature control and Nose–Hoover Langevin piston for pressure control (68,69). The particle mesh Ewald (PME) technique (70) was used for the electrostatic calculations. The van der Waals and short-range electrostatics were cut off at 12.0 Å with switch at 10.0 Å. Each system has two replicas of simulations.

#### Data analyses

We analysed the simulation trajectories using TCL scripts implemented in VMD (64) and plotted by our in-house Python scripts. Polar contacts within 3.6 Å were shown by Pymol (Schroödinger Inc.). We calculated the structural changes by RMSD and RMSF α carbon alignments of res. 5–41 and res. 55–81 regions on the crystal structure (PDBID: 3NR7) for site1 and site2 respectively. The intermolecular polar contacts were calculated between a hydrogen donor (nitrogen/oxygen/sulphur attached to a hydrogen) and an acceptor (oxygen) with a cutoff of 3.6 Å (thus, including the contacts in backbone and sidechain). We chose a medium cutoff of 6.5 Å for the hydrophobic contacts by carbon–carbon pairs (not including α carbon) of two intermolecular residues.

### PMF calculations

We calculated the PMFs of the dissociation processes of site1 (res. 2–47) and site2 (res. 50–82) using the adaptive biasing force (ABF) method (71,72) which has shown better efficiency compared with alternative methods (73,74). In preparation for the PMF calculations, we used steered molecular dynamics to generate a trajectory in which one monomer was slowly pulled away from the other monomer. From the pulling trajectory, we selected 9–10 windows in the span of 0–40 Å as the reaction coordinate; the distance between the backbone COM of two monomers is used as the collective variable. To analyse the numerical convergence of PMF, we examined the time sequential PMFs every 2 ns and observed changes as the simulation progresses. The difference between PMF curves became relatively small after 25 ns/replica. We computed the average value with the standard deviation (shown as the error bar) for each bin along the reaction coordinate from the last six PMFs.

### Microscale thermophoresis (MST)

H-NS C-termini (84–137) and (91–137) were N-terminally labeled with RED dye (NT-647) following the protocol from manufacturer (Nanotemper, Germany). Free dye was removed by passing through the Sephadex G-25 column and for Cy5 labeled DNA used in our experiments it was purchased directly from IDT company. Serial dilutions of unlabeled samples were mixed with 20 nM of NT-647 labeled proteins and Cy5 labeled DNA in buffer (50 mM Tris–HCl, 50 mM NaCl, 2 mM MgCl_2_, 0.005% Tween-20) and incubated for 30 min. The same samples were subjected to different levels of salinity (100, 250, 500 and 1000 mM of NaCl) and measured in the same way. 10 μl of sample were loaded into premium monolith NT capillaries and assays were carried out in a Nanotemper Monolith NT.015T. *K*_d_ values were determined by plotting the concentrations of unlabeled ligands against the changes in fluorescent thermophoresis signal, and the data was fitted using a single-binding-site (Graph Pad).

### Fluorescence anisotropy

The binding affinity of the Cy5 labeled 101 bp DNA with four binding sides (IDT, Belgium), toward H-NS_FL_ and H-NSΔs2 was determined using spectraMax i3 (Molecular Devices) plate reader. The Cy5-labeled DNA has excitation and emission wavelength of 649 and 666 nm respectively. Data are the average of independent experiments and error bars correspond to standard deviations at two different temperatures of 25 and 37°C. The buffer solution for assays consisted of 20 mM HEPES pH 7.5, 50 mM NaCl and 2 mM MgCl_2_. Measurements began at 50 μM of the protein, followed by successive 2-fold sample dilutions with buffer, until reaching the lowest protein concentration (390 nM) in the presence of 20 nM Cy5 labeled DNA. The *K*_d_ value for the DNA was fitted using a single-binding-site (Graph Pad). Binding studies were carried out using shorter 101 bp DNA, because the manufacturer could not synthesize fluorescently labeled longer DNA.

### Isothermal titration calorimetry (ITC)

H-NS N-terminal (2–57) and H-NS 91–137 C-terminal (91–137) region of H-NS were dialysed in ITC buffer (20 mM HEPES pH 7.5, 150 mM NaCl, 1 mM DTT). 200 μl of H-NS 2–57 N-terminus was placed in the cell at a concentration of 20 μM, whereas H-NS 91–137 C-terminus was kept at a concentration of 300 μM in the injection syringe. Titrations were performed at 25°C with an initial injection of 0.4 μl, followed by 25 injections of 2 μl. ITC was performed on MicroCal ITC200 from GE Healthcare and data was fitted using Origin Software.

### Nuclease assay

H-NS_FL_ and H-NSΔs2 at 20 μM final concentration were incubated with 1 μg lambda DNA (NEB) at 25 and 37°C in the presence of 5 units of DNAse (EMD Millipore) in a Thermocycler (Biorad) for 30 min. Samples were then mixed with 5 μl of 6× loading buffer (NEB) and resolved on a 0.7% agarose gel mixed with 1× Cybersafe (Lab technologies). Gels were run in TAE buffer at 110 V for 1 h and visualized using gel doc (Biorad).

### Circular dichroism spectroscopy

CD spectra of purified proteins of H-NS_FL_ and H-NSΔs2 and H-NS_2–57_ at final concentration of 200 μM in buffer containing 10 mM sodium phosphate pH 7.2, 50 mM NaCl, 2 mM MgCl_2_, was recorded using CD spectrophotometer (JASCO), wavelength ranging from 190 to 260 nm. The data were smoothened using inbuilt JASCO software. To determine the melting curve for H-NS_FL_, data were recorded with increase of temperature and plotted against the ellipticity at 225 nm using Graph Pad software.

### Differential static light scattering (DSLS)

DSLS measured by the Stargazer system (Harbinger Biotechnology and Engineering Corporation, Markham, Canada) was used to assess the thermal stability of H-NS in the presence of buffer with varying pH. 50 μM of H-NS_FL_ were overlaid with mineral oil in a clear bottom 384-well black plate (Corning), and heated from 20 to 85°C at 1°C/min; light scattering was detected by a CCD camera every 0.5°C in the presence of buffers of varying pH. Data were recorded from three independent experiments with standard deviation and tabulated.

## RESULTS

### *S. typhimurium* H-NS site1 does not change conformation with temperature, osmolarity or pH.

To test if environment sensing proceeds through a transition between parallel and antiparallel site1 structures, we compared small angle X-ray scattering (SAXS) pattern of *S. typhimurium* H-NS_2–57,C21S_ under a wide range of temperature, salt and buffer conditions. Under all conditions tested, including those under which the parallel NMR structure of *S. typhimurium* H-NS_1–57,C21S_ was determined (20°C, 300 mM NaCl, pH 7.0) (29), the SAXS patterns were identical, demonstrating that the conformation of H-NS_2–57,C21S_ did not change (Figure 2B, Supplementary Figure S1A, B). Accordingly, the SAXS-derived model-independent values for the maximum diameter (*D*_m_) and the radius of gyration (*R*_g_) did not change significantly across conditions and buffers tested (*D*_m_ were 74 ± 2 Å, and *R*_g_ were 21 ± 1.8 Å). These *D*_m_ and *R*_g_ values were in agreement with those calculated for the antiparallel H-NS_2–57,C21S_ model (*D*_m_ = 75 Å; *R*_g_ = 19.4 Å), but not with those of the parallel structure (*D*_m_ = 58 Å; *R*_g_ = 18.5 Å). Moreover, all SAXS-derived pair-wise distance distribution *P*(*r*) plots matched the *P*(*r*) calculated for the antiparallel model, but not the *P*(*r*) of the parallel model, because of distances in the range of 60–75 Å, characteristically present only in the larger antiparallel model (Figure 2C, D and Supplementary Figure S1C). Finally, the experimental SAXS patterns for all conditions fitted the SAXS pattern calculated for the antiparallel site1 dimer significantly better than those calculated for the parallel model [χ^2^_T = 10°C_: 2.22 (antiparallel); 7.56. (parallel). χ^2^_T = 42°C_: 4.20 (antiparallel); 11.62 (parallel) Figure 2E].

These data demonstrated that H-NS_2–57,C21S_ does not change conformation within a relevant range of conditions, and that its conformation is the antiparallel site1 structure. Thus, our data provided compelling evidence against a model where temperature, salt or acidity are sensed through a parallel-to-antiparallel structural transition of site1.

### Temperature, salt and pH dependency of site2 dimerization

We next used dynamic light scattering (DLS) to directly assess the effect of physiochemical parameters on H-NS multimerization in solution. DLS analyses of full-length *S. typhimurium* H-NS showed a gradual reduction in the hydrodynamic radius (*R*_h_; corresponding to the average molecule size), upon increase of the temperature from 5 to 42°C (Figure 2F). The higher the protein concentration, the greater were the *R*_h_, as expected for a concentration-dependent multimerization. Protein aggregates started to affect the DLS signals at temperatures above 42°C. Circular dichroism (CD) analysis confirmed that the secondary structure of H-NS was intact at 37°C and determined the melting temperature to be 48.5°C, in good agreement with an aggregation temperature of ∼52°C (Supplementary Figure S1D–F). H-NS with a deletion of site2 residues 58–82 (H-NSΔs2) still preserved the secondary structure content of the wild-type (Supplementary Figure S1D), but did not markedly change particle size with temperature (Figure 2F). Given that site1 structure and dimerization did not change between 10 and 40°C, these data suggested that temperature destabilized site2, which affected the capacity of H-NS to multimerize beyond site1-linked dimers. Accordingly, H-NSΔs2 would form temperature-insensitive site1-linked dimers. However, due to the small size of H-NSΔs2, the DLS data were too noisy to experimentally verify this prediction (Figure 2F).

To increase the particle size and hence reduce the DLS signal noise, we used H-NS constructs that had an N-terminal mCherry fusion (^mCh^H-NS). This modification did not change the apparent temperature dependency of the DLS signal of full-length H-NS, and hence did not alter its multimerization behavior (compare Figure 2F and G**)**. ^mCh^H-NSΔs2 did not change size with temperature, and allowed confident measurement of its *R*_h_ of 5 nm (Figure 2G, H and Supplementary Figure S2A). This *R*_h_ was in good agreement with the *R*_h_s calculated from 3D structural models based on H-NS dimers linked through the anti-parallel site1 arrangement (*R*_h_s for ^mCh^H-NS, ^mCh^H-NSΔs2 and H-NSΔs2 dimers were 5.08, 4.90 and 3.25 nm, respectively, whereas the *R*_h_s for these monomers were 3.49, 3.34 and 2.47 nm, respectively; Supplementary Figure S2B). Based on our models, the experimental *R*_h_ values of 14–20 nm for the higher-order multimers might correspond to 4–8 site2-linked dimers (Supplementary Figure S2B). The *R*_h_ versus temperature [*R*_h_(*T*)] plots of ^mCh^H-NS fitted well with a sigmoidal function, assuming the *R*_h_ = 5 nm of H-NSΔs2 as the lower plateau (Supplementary Figure S3A, B). Sigmoidal behavior is observed in cooperative protein unfolding (43), suggesting that heat reduces multimerization by unfolding site2.

We also tested the effects of osmolarity and pH. Increasing the NaCl concentrations from 150 to 500 mM lowered the *R*_h_ (*T* = 5°C) markedly from ∼20 to ∼15 nm at pH 7, and hence weakened H-NS multimerization (Figure 2G). The overall shape of the *R*_h_(*T*) plots did not change with salinity and the inflection points (*x*_0_) of the fitted sigmoids varied little (Supplementary Figures S2A, S3A and B). Thus, salt changed the amplitude of the temperature-dependency but not the overall response dynamics. Lowering the pH from 8 to 6 destabilized multimerization only at high salinity, and appeared to promote protein aggregation *in vitro* at 150 mM NaCl (Figure 2H, Supplementary Figures S2A, S3A and B).

### Potential of mean force (PMF) calculations distinguish the stability of site1 and site2

**Figure 3.**
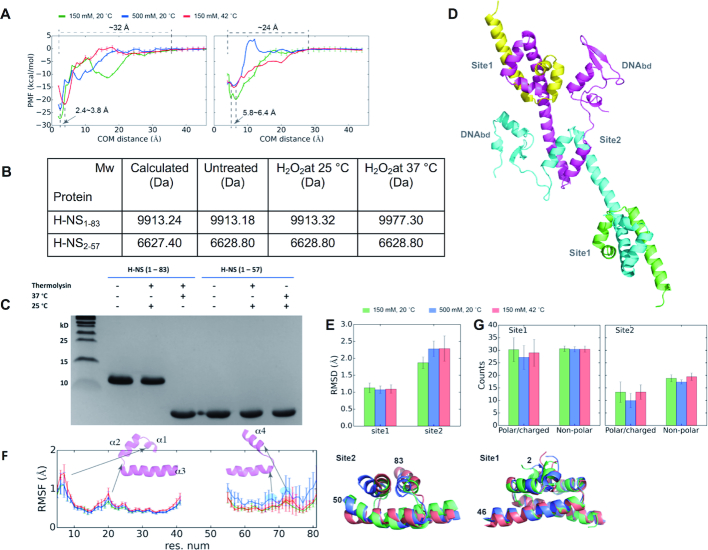
Site2, but not site1, of S. typhimurium H-NS is sensitive to changes in temperature and salinity. (**A**) PMF plots of site1 (left) and site2 (right) dimers along the pulling direction using COM distance between two monomers as the collective variable. The dissociation state of the monomers is set as the reference (0.0 kcal/mol) for each calculation. We averaged the data with SDs as error bars from six PMFs; the small standard deviations (SDs) indicate the convergence of the PMF. (**B**) Molecular weight determination of H-NS_1–83,C21S_ or H-NS_2–57,C21S_ at different temperatures in the presence or absence of hydrogen peroxide (H_2_O_2_). Calculated Mw for unmodified proteins includes leftover of cleavage from tags (H-NS_1–83,C21S_: residues GPLGS; H-NS_2–57_: GS). (**C**) Proteolytic cleavage of H-NS_1–83_ or H-NS_2–57_ with thermolysin at different temperatures. (**D**) A final snapshot of the full-length H-NS dimer (*centre*), and superimposed site1 (*top*) and site 2 (*bottom*) structures under regular, salt, and heat conditions taken from 200 ns MD simulations. (**E**) Histogram of Cα RMSD of site1 and site2 dimers, with the *S. typhimurium* H-NS crystal structure (PDBID: 3NR7) as the reference. We superposed residues 5–41 and residues 55–81 for the RMSD calculations of site1 and site2, respectively. (**F**) Plot of average residue fluctuation (RMSF) for site1 and site2 with respect to the crystal structure (PDBID: 3NR7), presented along the H-NS sequence. Prominent features the high-temperature and high-salinity conditions are highlighted in red and blue, respectively. (**G**) Histograms of the polar(H-bond)/charged(ionic) contacts and of non-polar(hydrophobic) contacts across protein chains of site1 and site2 dimers. The data in (E) and (G) were averaged with SDs as error bars from all replica simulations.

Our results showed that site1 is robust to physiochemical changes, and suggested that these parameters act through unfolding of site2. To assess the energetics of site1 and site2 dimerization, we performed PMF calculations of the site1 and site2 dimer dissociation processes under three conditions: (i) 150 mM NaCl at 20°C (a regular condition), (ii) 500 mM NaCl at 20°C (at high salinity) and (iii) 150 mM NaCl at 42°C (at high temperature) (Figure 3A, Supplementary Figures S4 and S5). At the energy minimum, site1 has a shorter centre-of-mass (COM) distance (2.4–3.8 Å) than site2 (5.8–6.4 Å), and the dissipation distance to the dissociated state (0.0 kcal/mol) was also longer for site1 (∼32 Å) than for site2 (∼24 Å) (Figure 3A). Both observations are consistent with site1 being more compact and having more residues and intermolecular contacts, in agreement with their crystallographic structures.

The difference between the energy minimum of the dimeric state and the energy of the dissociated state represents the dimeric thermodynamic stability. Under the regular condition, this energy difference was 27.3 ± 0.4 and 19.8 ± 0.9 kcal/mol for site1 and site2, respectively. When exposed to high salt or heat, the energy difference decreased, respectively, to 23.7 ± 0.6 and 21.7 ± 0.7 kcal/mol for site1, and to 14.4 ± 0.2 and 15.1 ± 0.2 kcal/mol for site2 (Figure 3A). At high salinity, we observed a kinetic energy barrier for site2 of about ∼3.5 kcal/mol upon the dissociated states, corresponding to a state when the two monomers loosely associated by weak polar/charged contacts (Figure 3A, Supplementary Figure S5C). Together, these analyses supported that temperature and salinity affect the stability of both dimeric structures. However, site2 has a markedly lower thermodynamic stability than site1. Thus, under heat strain the site2 dimer would dissociate much earlier than site1.

### Direct *in vitro* evidence for site2 unfolding

We next performed two sets of experiments to test whether site2 unfolds prior to other H-NS regions. First, H-NS_1–83,C21S_ (which encompasses site1 and site2) and H-NS_2–57,C21S_ (with site1 only) were subjected to oxidation by hydrogen peroxide (H_2_O_2_) at 25 and 37°C. Under these conditions, solvent-accessible cysteines and methionines are oxidized, which results in an increase in molecular mass that can be monitored using mass spectrometry (MS). At 25°C there was no change in molecular weight of both proteins, implying that the methionines of site1 (M1, M29) and of site2 (M64, M74) were protected within the three-dimensional fold of these domains (Figure 3B). At 37°C, the molecular weight of H-NS_1–83_ increased by 64 daltons, corresponding to four oxygens, whereas H-NS_2–57_ remained unchanged. This observation is consistent with oxidation of M64 and M74 to methionine sulfones due to unfolding of site2 at 37°C, while site1 remained structurally intact.

Next, we subjected H-NS_1–83,C21S_ and H-NS_2–57,C21S_ to proteolytic cleavage with thermolysin at 25 or 37°C. Thermolysin preferentially cleaves at the N-terminus of the hydrophobic residues leucine, isoleucine, valine, methionine, phenylalanine and alanine of solvent accessible (unstructured) regions; this cleavage is hindered if the proceeding residue is acidic. At 25°C, neither of the proteins showed clear signs of cleavage, in agreement with internal cleavage sites being protected by a native structuring of site1 and site2. At 37°C, H-NS_2–57,C21S_ remained apparently uncleaved, whereas H-NS_1–83,C21S_ was cleaved to yield a fragment of about the same size as H-NS_2–57,C21S_ (Figure 3C). MS analysis of the cleavage product provided a Mw of 6196.9 Da, implying the resulting fragment is H-NS_2–57_ (calculated Mw is 6196.0 Da). These data demonstrated that heat specifically unfolded site2, while the tertiary (dimeric) structure of site1 of H-NS remained unchanged. Although heat unfolded site2, the helical structure of α3 was preserved until at least V51 (i.e. the next thermolysin cleavage site N-terminal to L58).

### Molecular dynamics (MD) simulations reproduce the stability of the site1 N-terminal dimer

We next used MD simulations to investigate the reaction of H-NS to temperature and salinity at atomic detail. First, we simulated the dynamics of a multimeric site1 and site2-containing full-length *S. typhimurium* H-NS construct (Figure 3D) under three conditions: (i) 150 mM NaCl at 20°C (a regular condition), (ii) 500 mM NaCl at 20°C (at high salinity), and (iii) 150 mM NaCl at 42°C (at high temperature) (Supplementary Figures S4 and S6; see Methods). For site1, the small root-mean-square deviation (RMSD = ∼1 Å, Figure 3E) and the small root-mean-square fluctuation (RMSF = ∼1.5 Å, Figure 3F) per residue implied only subtle conformational changes; as the temperature increases, fluctuations mainly increased in the N-terminus of α1 (especially at K6 and I7) and the loop between α2 and α3 (around E20) (Figure 3E). An increase in salinity only mildly raised the RMSF around E20, as a result of decreased intermolecular polar/charged contacts (Figure 3G). Thus, site1 appeared robust toward heat and salt in our simulations, mirroring the high *in vitro* stability of site1.

The overall RMSD of site2 was larger than site1 in the regular condition, and further increased in the high temperature or salt conditions, concomitant with increasing fluctuations (Figure 3E, F). The reduced stability of site2 is explained by it forming fewer polar/charged and hydrophobic interactions at the dimer interface compared to site1 (Figure 3G). Under all conditions, the N-terminal half of α4 (residues 71–75) showed above-average RMSF. The same region responded with highest RMSF increases to heat or salinity (highlighted in red and blue, respectively, in Figure 3F). This region contains D71 and E74; D71 is capping α4, and both D71 and E74 form intramolecular ionic bond pairs (D71:R90’ and R54:E74’, where the apostrophe denotes the second chain. Supplementary Figure S7A). Additionally, at high salinity we observed increased fluctuations for residues 67–70 (second blue area in Figure 3F). This area included D68, which forms an ionic bond with K57’ (Supplementary Figure S7A). *In silico* mutation of D71 and E74 into alanines reduced the effect of salt, while increasing or maintaining the amplitude of conformational changes in the normal and high-temperature conditions (Supplementary Figure S7B), in agreement with a role of these residues in mediating the heat and salinity response.

A 700-ns long simulation of a full-length *S. typhimurium* H-NS dimeric construct under excessive temperature (147°C) further confirmed that the dimeric site1 structure including helix α3 remained relatively stable whereas the helix α4 of site2 turned into a loose coil (Supplementary Figure S7C). Together, our MD simulations designated site2 as the agent of heat and salt sensing and suggested that the N-terminal half of α4 and the proceeding loop play a key role in translating environmental changes into site2 destabilization.

### Site2 unfolding enables an interaction between H-NS N- and C-terminal regions

**Figure 4.**
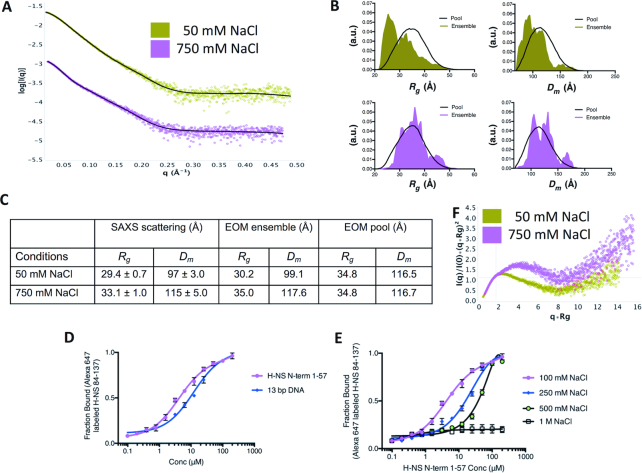
H-NSΔs2 adopts a relatively compact conformation in vitro. (**A**) Experimental H-NSΔs2 SAXS patterns recorded at 50 mM NaCl (green) and 750 mM NaCl (pink). Calculated SAXS pattern from ensemble models are shown in black. The corresponding Guinier plots are shown in Supplementary Figure S8. (**B**) Histogram of *R*_g_ and *D*_m_ for pool (black line) and selected ensemble models (olive for 50 mM NaCl data, and pink for 750 mM NaCl data). a.u: arbitrary units. The corresponding ensemble models are shown in Supplementary Figure S8. (**C**) *R*_g_ and *D*_m_ values calculated from (A) and (B) are tabulated. (**D**) Interactions of fluorescently labeled H-NS_84–137_ with H-NS_1–57_ and 13bp DNA determined using MST. (**E**) Influence of NaCl on the H-NS_84–137_:H-NS_1–57_ interaction determined using MST. Data in (D, E) are means ± S.D., *n* = 3.

We used SAXS to gain further experimental insights into the molecular conformation of H-NS in its dimeric high-temperature state. Because SAXS measurements at ≥40°C on full-length H-NS contained aggregates and ‘contaminations’ with higher-order dimers, we used constitutively dimeric H-NSΔs2 (at 10°C) for this analysis (Figure 4A, Supplementary Figure S8). To probe the relative orientation of the C-terminal domains with respect to the N-terminal dimer, we used an ensemble-optimization method (EOM) approach. This approach first creates a pool of stereochemically possible structural models, given the flexible linker between the N-terminal and C-terminal domains, and then selects a multi-model ensemble that best fits the SAXS data (44).

Based on our thermolysin cleavage results, we used as rigid bodies in EOM the dimeric H-NS_1–54_ model (based on PDB 3NR7) and the folded C-terminal DNAbd_94–137_ (based on 2L93). We observed that the histogram of the selected structural ensembles was markedly skewed toward smaller *D*_m_ and *R*_g_ values as compared to the pool of possible structures (χ^2^ fit = 1.05; Figure 4, Supplementary Figure S8). Hence, the C-terminal DNAbds of H-NSΔs2 were closer to the site1 dimeric core than expected if they were freely protruding in the solvent at the end of the flexible site2-DNAbd linker.

**Figure 5.**
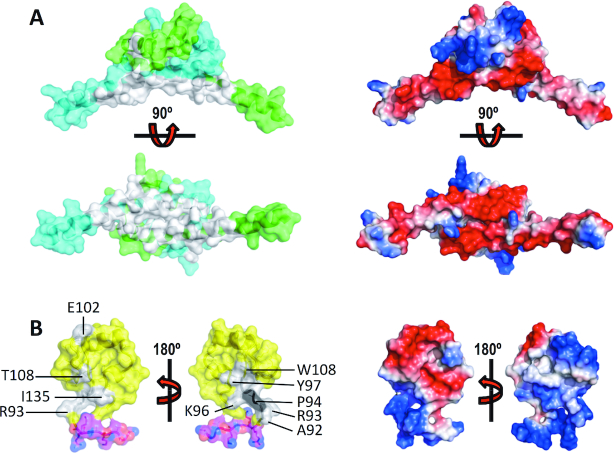
The H-NS C-terminal region binds to the N-terminal domain. (**A**) *Left:* Chemical shifts (grey) mapped on H-NS_2–57_ chains A (green) and B (cyan). *right:* electrostatic surface of H-NS_2–57_. Top and bottom panels show 90° views. (**B**) *Left:* Chemical shifts (grey) mapped on H-NS_84–137_. Proline 94 is shown in black. Yellow: DNAbd; magenta: linker residues 84–90. *Right:* electrostatic surface of H-NS_84–137_. For each representation, 180° views are shown, as indicated.

This observation could be caused by intramolecular interactions between the N-terminal and C-terminal domains. Therefore, we tested whether the N-terminal dimer interacts with the C-terminal region of H-NS. Microscale thermophoresis (MST) showed that H-NS_2–57_ bound to H-NS_84–137_ with a dissociation constant *K*_d_ of 4.1 ± 0.4 μM (Figure 4D). This association was significantly stronger than the association of H-NS_84–137_ with a 13 bp DNA (31) *K*_d_ of 14.5 ± 1.2 μM. The direct interaction between these H-NS fragments was confirmed using NMR, by titrating ^15^N-labeled H-NS_2–57_ with unlabeled H-NS_84–137_, and *vice versa*. On both domains, the interaction resulted in relatively small chemical shift changes and line broadening, indicating intermediate exchange (Supplementary Figure S9A, B). Based on available chemical shift assignments (31,45) we were able to identify residues L8 (on α1), L14, R15, A16 (on α2), L23, E27, L30, E31, L33, E34, V35, V37, N38, E39, R41, E42, E44, S45 and A46 (on α3) on H-NS_2–57_ as being involved in the association (Figures 1A, 5A and Supplementary Figure S9A, B). When mapped onto the molecular surface of dimeric H-NS_2–57_, these residues formed a well-defined surface-exposed cluster on α3 (Figure 5B), On H-NS_84–137_ only a few backbone amide signals were gently affected by the addition of unlabeled H-NS_2–57_ (A92, R93, A95, K96, Y97, E102, T108, I135) (Figures 1A, 5A and Supplementary Figure S9B). Except for E102, the affected DNAbd residues form a surface patch close to the linker region (Figure 5B). The linker residues 84–91 were broadened on the spectrum, implying that high intrinsic dynamics of this region result in amide exchange with the solvent.

On H-NS_2–57_ mostly negatively charged residues were affected by the presence of H-NS_84–137_, suggesting that the interaction between the two domains is largely based on ionic interactions between negative charges on H-NS_2–57_ and positive linker residues that leave the DNAbd domain only loosely connected (46,47). In confirmation of the importance of ionic interactions, the affinity between H-NS_2–57_ and H-NS_84–137_ was strongly diminished by increasing buffer salinity (Figure 4E). Additionally, SAXS measurements on H-NSΔs2 at high salt (750 mM NaCl) showed an increased particle size (Figure 4A, C, Supplementary Figure S8), and EOM produced ensemble models (χ^2^ fit = 1.03) of the same dimensions as the pool, demonstrating a loss of intramolecular contacts between N and C-terminal domains (Figure 4B, C). In agreement, normalized Kratky plots of the high-salt data were indicative of increased flexibility and greater separation of the domains, as compared to the low-salt condition (Figure 4F).

Moreover, a C-terminal construct without the linker residues (H-NS_91–137_) failed to associate with H-NS_2–57_ in NMR, MST and isothermal titration calorimetry (ITC) experiments, confirming the key role of the charged linker residues for the interaction (Supplementary Figure S9C–E). Importantly, when H-NS adopts a multimeric conformation with an intact site2 dimer (as in Figure 1B), the linker region is located >25–30 Å away from the interacting N-terminal residues, which is too far to enable their interaction. This interaction would stereochemically only be possible upon unfolding of site2, as promoted at high temperatures or in the H-NSΔs2 mutant.

### N/C-interaction blocks DNA binding H-NS_2-57_

**Figure 6.**
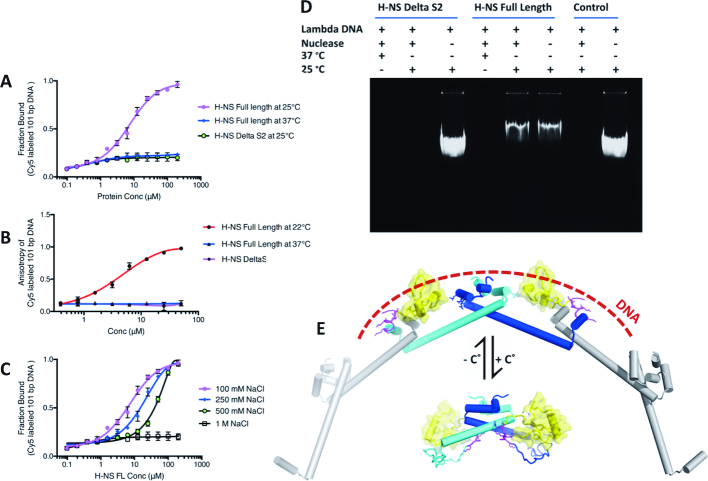
H-NS:DNA interactions are incompatible with the H-NS N/C association. Interactions between H-NS_FL_ and H-NSΔS2 with 101bp DNA were determined using (**A**) MST and (**B**) fluorescence anisotropy. (**C**) Influence of NaCl on the interaction between H-NS_FL_ with 101bp DNA, determined using MST. Data in (A–C) are means ± S.D., *n* = 3. (**D**) Nuclease assay of lambda DNA in the presence of H-NS_FL_ and H-NSΔS2 under different conditions. (**E**) Proposed temperature switch mechanisms. *Top:* Under conditions where site2 dimers are stable, H-NS will multimerize. In this conformation, the linker residues (magenta), DNAbd (yellow) and positive charges on site1 (stick representation) of linked H-NS molecules can cooperate to achieve a strong association with DNA (indicated as dashed line). *Bottom:* Under conditions where site2 unfolds, the C-terminal linker and DNAbd associate with the N-terminal site1, leading to an autoinhibited dimeric conformation incapable of DNA binding and of formation of higher-order multimers. Although based on experimental data, the positions of the acidic linker and DNAbd are speculative.

Although the linker residues 84–90 are not part of the folded DNAbd, they are required for strong DNA interactions (30,32). Given that H-NS_84–137_ bound stronger to H-NS_2–57_ than to DNA (Figure 4D, E), we tested whether the association between the N- and C-terminal residues, which can only occur when site2 is molten, blocks binding of H-NS to DNA. For these experiments, we used a short cy5-labeled 101 bp DNA with four consensus H-NS binding motifs designed to allow binding of an H-NS tetramer (see Methods). At room temperature, H-NS bound to this 101 bp DNA with micromolar *K*_d_s (7.4 ± 0.9 μM in MST; 10.2 ± 1.2 μM in fluorescence anisotropy experiments) (Figure 6A, B). These affinities agreed with previous studies (48). Increasing NaCl concentrations decreased the affinity of the H-NS:DNA interaction (Figure 6C), as expected from a charge-mediated association between the linker and DNA. We also observed that H-NS did not bind DNA at 37°C, whereas H-NSΔs2 failed to associate with DNA already at 25°C (Figure 6A, B). Given that the isolated DNAbd_84–137_ measurably associated with DNA (Figure 4D and (31)), the absence of DNA interactions of H-NSΔs2 (which contains two DNAbd_84–137_ regions) demonstrated that the N:C-terminal interaction effectively blocked DNA binding in absence of a folded site2 dimer. Accordingly, in a nuclease assay H-NS protected the 48502 bp lambda DNA from cleavage at 25°C, but not at 37°C, whereas H-NSΔs2 failed to protect DNA already at 25°C (Figure 6D). Together, these data reveal a mechanism where temperature modulates the stability of site2 dimers, and hence controls switching between a multimeric DNA-binding conformation and a dimeric autoinhibited conformation (see Figure 6E for a schematic representation).

## DISCUSSION

H-NS is an important regulator for function and fitness of Gram-negative bacteria, including human pathogens and multidrug resistant bacterial strains. Despite decade-long efforts of many groups, the structural mechanism by which H-NS translates environmental changes into changes in gene expression remained poorly understood. Indeed, the size, dynamic multimerization and intrinsic flexibility of H-NS precludes study of this mechanism by X-ray crystallography, NMR or cryo-electron microscopy. To determine the molecular mechanism for environment-sensing by H-NS, we therefore combined experimental methods that directly monitor structural changes on different resolution scales (SAXS, DLS, NMR) with dynamics and molecular detail from MD simulation and homology modeling.

We showed *in vitro* and *in silico* that the N-terminal site1 dimerization domain of *S. typhimurium* H-NS does not change conformation in response to changes in physiochemical parameters, conversely to a previously proposed model. H-NS site1 preserved an antiparallel dimeric conformation under all conditions tested, in agreement with previous MD simulations (28) and crystallographic or NMR analyses that showed this fold at a range of temperatures (16, 20, 42°C), pHs (6.2, 6.8 and 7.5), salt concentrations (0 to 250 mM NaCl; and possibly much higher in reference (26)), and using various sequences [*S. typhimurium* H-NS_1–83,C21S_, *S. typhimurium* H-NS_1–46_, *E. coli* H-NS_1–64_, *E. coli* H-NS_1–46_ (both containing C21) and *Vibrio cholera*e VicH_1–48_] (25,26,35,42). Conversely, the site2 fold, and hence dimerization, was sensitive to temperature and salinity *in silico* and *in vitro*. Our MD simulations revealed that α4, in particular the interactions of residues D71 and E74, plays a key role in translating temperature and salinity changes into site2 (de)stabilization. The *in vitro* temperature-dependency of H-NS multimerization fitted a model of cooperative protein domain unfolding. Together our data provide compelling evidence that environment-sensing by H-NS proceeds through (un)folding of site2 rather than through conformational switching of site1.

We further demonstrated that unfolding of site2 affects DNA interactions in two ways: Firstly, by precluding a synergistic DNA interaction of multiple site2-linked H-NS dimers, and secondly by promoting a charge-based autoinhibitory ‘closed’ conformation which involves the C-terminal acidic linker and thus blocks its required contributions to DNA binding. Temperature had the strongest influence on site2 unfolding. An increase in salinity had several effects: it mildly destabilized H-NS multimerization, and it markedly weakened the charge-based interactions between (i) the C-terminal region and DNA, and (ii) between the H-NS N- and C-terminal regions. Entereobacteria cannot control their temperature, but have strong mechanisms for maintaining their cytoplasmic salinity. While the biological relevance of the observed salt effects is therefore debatable, the high-salt conditions helped us unravelling the molecular autoinhibition mechanism of H-NS.

The autoinhibitory H-NS form established herein is reminiscent to the ‘closed’ H-NS form that van der Valk *et al.* proposed based on MD simulations (41). However, their closed model is promoted by an unfolding of helix α3 residues 42–50 (in absence of magnesium ions), whereas ours is based on (thermal) unfolding of site2. We did not observe any evidence for such α3 flexibility in our thermolysin assay (which would have resulted in a cleavage N-terminal to alanines 46–49). And while we occasionally observed bending movements of the middle region of α3 in some of our simulations, these movements were random (i.e. not linked to heat or salinity) and did not lead to an unfolding of α3 required to bring the N- and C-terminal domains together. The differences in MD can be attributed to different starting models; we used a full-length model that also contained an intact site2 dimer (corresponding to the multimerized low-temperature form of H-NS; Figure 3D), whereas van der Valk *et al.* simulated H-NS with non-dimerized site2 regions. Although we cannot rule out contributions of α3 ‘buckling’, it is clear from our data that site2 melting is the driving force for the observed thermal disruption of H-NS multimers.

H-NS is part of a bacterial regulatory network that also includes multiple other factors, such as StpA and Hha (49,50). Therefore, the effects we observed for H-NS multimerization and DNA binding will be affected *in vivo* by the presence of other biomolecules. For example, the co-regulatory protein Hha binds to a surface on H-NS that partially overlaps with the binding site of the C-terminal region identified in our study (42,51) (Supplementary Figure S9F), suggesting that repression enhancement by Hha might partly result from interference with the autoinhibitory H-NS closed conformation. Understanding how all exogenous and endogenous factors combine to modulate the effects of H-NS will be an interesting task for future research.

Collectively, our results elucidate the molecular mechanism by which H-NS translates environmental changes into adapted gene expression. Given the pleiotropic control that H-NS exerts over gene expression, modulation of site2 dimerization or of autoinhibitory interactions by small molecules might provide a means to control toxicity of pathogenic bacteria that are resistant to current drugs.

## Supplementary Material

Supplementary Data
