# Supplementary material for: H-NS uses an autoinhibitory conformational switch for environment-controlled gene silencing
Source: Nucleic Acids Res. 2018 Dec 28;47(5):2666–80. doi: 10.1093/nar/gky1299 (PMC6411929; doi:10.1093/nar/gky1299)
Supplement: Supplementary Data [file gky1299_supplemental_files.pdf]

SUPPLEMENTARY INFORMATION

Figure S1: Biophysical assessment of the response of H-NS to changes in physiochemical parameters.

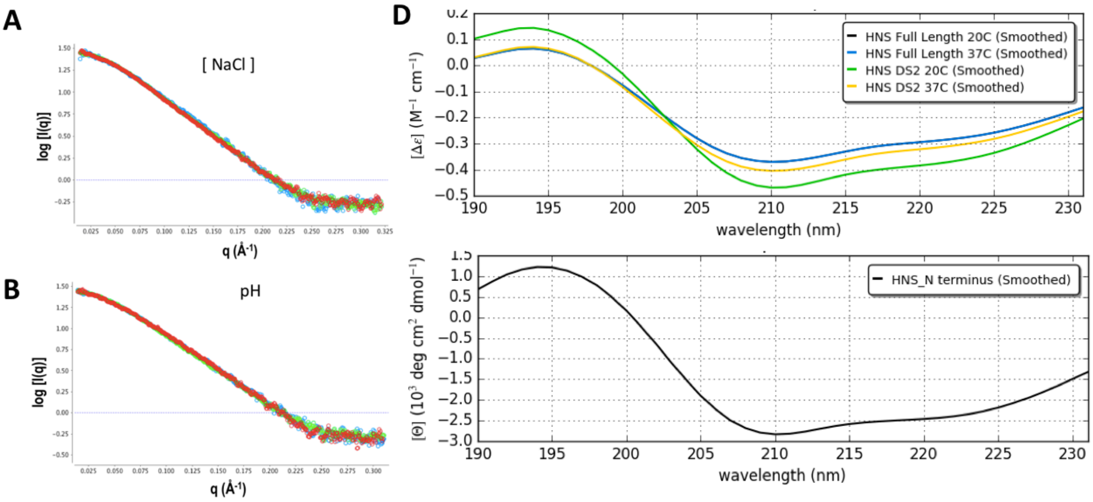

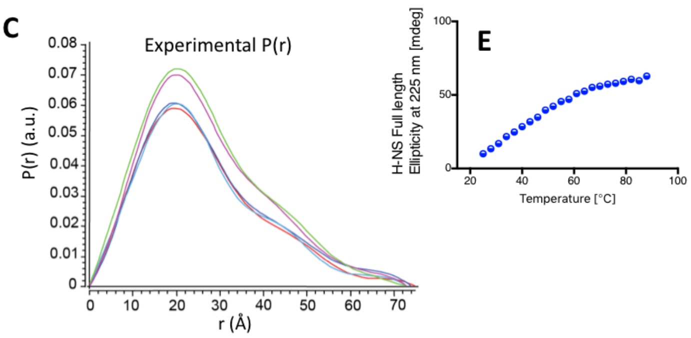

F

Buffer and pH	H-NS$_{FL}$ T_{agg}
MES Buffer, pH 5.5	51.7 ± 0.40 ∘C
PIPES Buffer, pH 6.5	52.77 ± 0.59 ∘C
HEPES Buffer, pH 7.5	53.36 ± 0.25 ∘C
TRIS Buffer, pH 8.5	52.62 ± 0.30 ∘C

(**A**) Superimposition of SAXS pattern collected in 1 mM EDTA, 20 mM phosphate buffer, pH 7.0 and 50 mM NaCl (red), 300 mM NaCl (green) or 500 mM NaCl (blue). (**B**) Superimposition of SAXS pattern collected in 1 mM EDTA, 300 mM NaCl, 20 mM phosphate buffer, pH 5.0 (red), pH 7.0 (green) or pH 9.0 (blue). (**C**) SAXS-derived *p(r)* for *S. typhimurium* H-NS$_{2\text{-}57,C21S}$ at 50 mM NaCl, pH 7.0 (magenta), 300 mM NaCl, pH 7.0 (green), 500 mM NaCl, pH 7.0 (red), and for 300 mM NaCl, pH 5.0 (blue), 300 mM NaCl, pH 9.0 (cyan). (**D**) (*top*) CD spectra for H-NS FL and H-NSΔs2 at 25 and 37∘ C. (*bottom*) CD spectra for H-NS$_{2\text{-}57,C21S}$ at 25 ˚C. Estimated secondary structure contents are: Full-Length (20 ˚C) α: 91.03; β: 0.3%. Full-Length (37 ˚C) α: 90.66; β: 0.3%. H-NSΔs2 (20 ˚C) α: 92.37; β: 0.26%. H-NSΔs2 (37 ˚C) α: 92.37; β: 0.26% (established using K2D3; Perez-Iratxeta C, Andrade-Navarro MA. (2007). K2D2: estimate of protein secondary structure from circular dichroism spectra. BMC Struct Biol, 8-25). (**E**) Ellipticity of H-NS measured using CD at various temperatures. (**F**) Aggregation temperature T*agg* of H-NS measured using DSLS at various pH. Data are mean ± S.D, *n* = 3.

Figure S2: Monitoring and calculating the size of solute S. typhimurium H-NS.

A

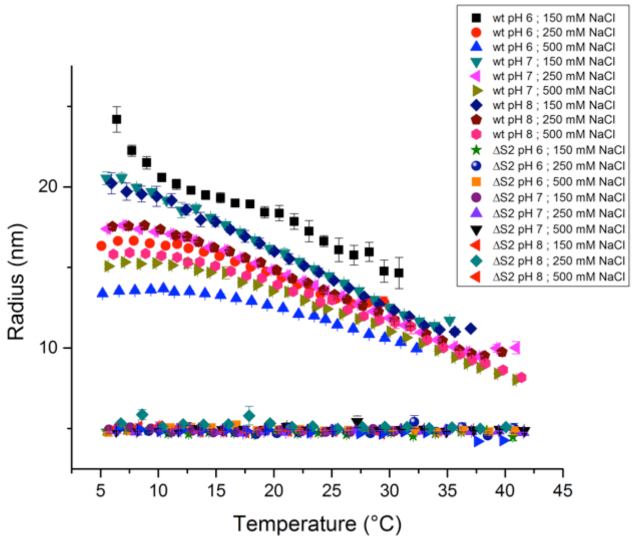

B

Protein	Mol Wt (Da)	Rh (DLS) (nm)	Rg (HydroPro) (nm)	Dt (cm^2/s)	Rh (HydroPro) (nm)
^{mChe}H-NS Dimer	86229	5 ± 0.2	5.00	4.23E-07	5.08
^{mChe}H-NS ΔS2 Dimer	80454		4.96	4.38E-07	4.91
^{mChe}H-NS Dimer x2	172458		7.86	2.43E-07	8.84
^{mChe}H-NS Dimer x3	258687		10.00	1.90E-07	11.31
^{mChe}H-NS Dimer x4	344916		13.60	1.45E-07	14.81
^{mChe}H-NS Dimer x6	517374		17.60	1.16E-07	18.52
H-NS ΔS2 Dimer	29875	3 ± 1	3.22	6.60E-07	3.26
H-NS ΔS2 Monomer	14946		2.42	8.69E-07	2.48
H-NS Dimer	30532		3.73	6.16E-07	3.49
H-NS Monomer	15266		2.54	8.18E-07	2.63
^{mChe}H-NS Monomer	42002		3.09	6.14E-07	3.50
^{mChe}H-NS ΔS2 Monomer	38999		2.87	6.41E-07	3.35
H-NS ΔS2 Close Conf. Dimer	29875		2.40	7.53E-07	2.86
H-NS ΔS2 Close Conf. Monomer	14946		2.01	9.28E-07	2.32

(**A**) DLS measurements of R_H as a function of temperature for ^{mCh}H-NS wt and ^{mCh}H-NSΔs2 at pH and NaCl concentrations as labeled. Data are means ± S.D, n = 3. (**B**) Experimental *vs*. calculated $R_{H.}$ D_t: translational diffusion coefficient. Close Conf: Rh values calculated for SAXS-derived structural models where the DNAbd was close to the site1.

Figure S3: DLS observation of the R_H under various temperatures and buffer conditions.

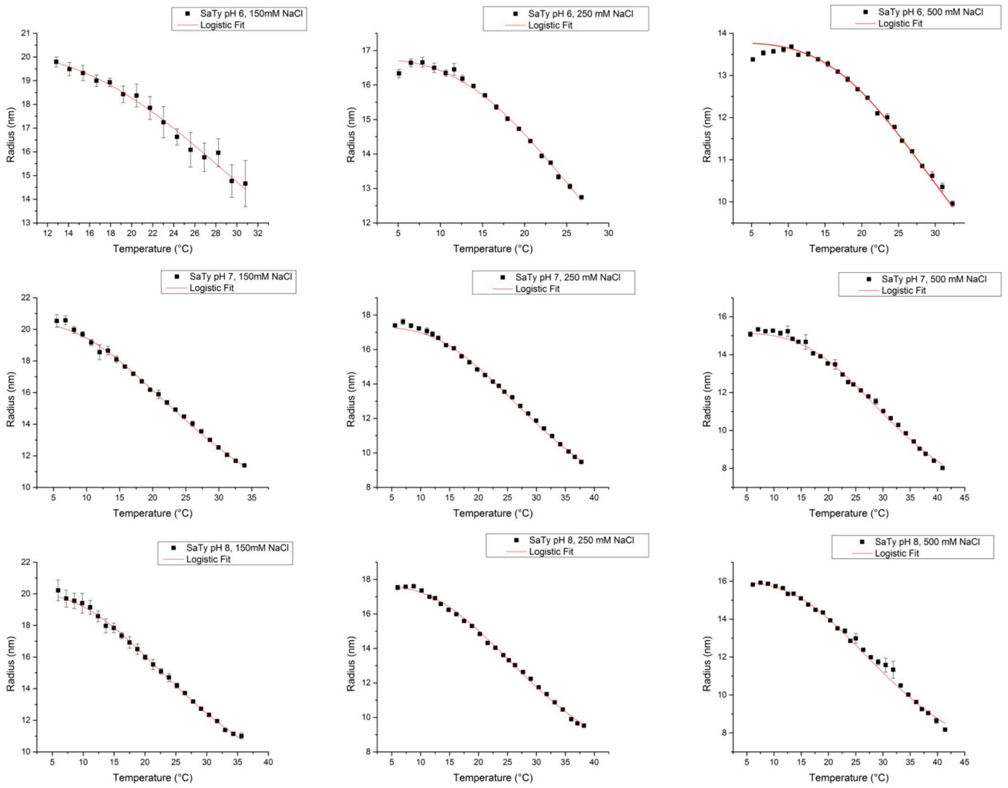

(**A**) Data for *S. typhimurium* ^{mCh}H-NS were fitted with a logistic fit assuming the lower plateau of 5nm. Data are means ± S.D, n = 3.

	S. typhimurium ^{mCh}***H-NS***		
pH	6	7	8
mM NaCl			
150	**A1** 20.3 ± 0.2<br>**x0** 35.6 ± 0.8<br>**S** -0.35<br>**T5** 14.5 ± 1<br>**T95** 87.6 ± 9	**A1** 20.4 ± 0.2<br>**x0** 29.3 ± 0.3<br>**S** -0.33<br>**T5** 9.1 ± 0.3<br>**T95** 95 ± 2	**A1** 20 ± 0.2<br>**x0** 29.8 ± 0.3<br>**S** -0.32<br>**T5** 9.6 ± 0.3<br>**T95** 82.4 ± 1.4
250	**A1** 16.8 ± 0.1<br>**x0** 33 ± 0.4<br>**S** -0.26<br>**T5** 12.2 ± 0.5<br>**T95** 89.7 ± 4.7	**A1** 17.3 ± 0.1<br>**x0** 31.8 ± 0.2<br>**S** -0.3<br>**T5** 12.3 ± 0.3<br>**T95** 82.4 ± 1.4	**A1** 17.7 ± 0.1<br>**x0** 31.4 ± 0.2<br>**S** -0.3<br>**T5** 11.6 ± 0.3<br>**T95** 85 ± 1.9
500	**A1** 13.8 ± 0<br>**x0** 34.6 ± 0.4<br>**S** -0.21<br>**T5** 14.6 ± 0.4<br>**T95** 82.1 ± 4.2	**A1** 15.1 ± 0.1<br>**x0** 32.9 ± 0.3<br>**S** -0.27<br>**T5** 14.4 ± 0.5<br>**T95** 75.5 ± 1.6	**A1** 16 ± 0.1<br>**x0** 32.5 ± 0.2<br>**S** -0.27<br>**T5** 12.8 ± 0.2<br>**T95** 83 ± 1.5

Summary of values fitted with a sigmoid function, with a lower plateau fixed at 5nm.
Values are indicated as the upper plateau (A1), the inflexion point (x0), the slope (S), the EC5 (T5) and EC95 (T95). Curves were fitted with a sigmoid function (logistic fit) given by y= (A1-A2 / (1+ (x/x0)^p)) +A2. A2 was fixed at 5nm. Errors are given as Standard Error; n = 3.

(**B**) Table of fitted values based on Fig. S3A.

Figure S4: Summary of all-atom MD simulations and PMF calculations.

Sys.	Model	PDBID	Num. of atoms	Box dimension (nm^3)	[NaCl] (mM)	T (˚C)	Simulation length (ns)
S. typhimurium [a]							
1	full-length (res. 2-137)	3NR7	102,953	13×9×10	150	20	200, 200
2	full-length (res. 2-137)	3NR7	102,101	13×9×10	500	20	200, 200
3	full-length (res. 2-137)	3NR7	102,101	13×9×10	150	42	200, 200
4	full-length (res. 2-137)	3NR7	99,000	13×9×10	150	147	700 (on Anton)
S. typhimurium with D71A and E74A mutations							
5	full-length (res. 2-137)	3NR7	106,226	13×10×10	150	20	200, 200
6	full-length (res. 2-137)	3NR7	105,346	13×10×10	500	20	200, 200
7	full-length (res. 2-137)	3NR7	106,226	13×10×10	150	42	200, 200
PMF calculations							2,820
8	Site1 dimer (res.2-47)	3NR7	18,172	6×6×6	150	20	470
9	Site1 dimer (res.2-47)	3NR7	18,172	6×6×6	500	20	470
10	Site1 dimer (res.2-47)	3NR7	18,172	6×6×6	150	42	470
11	Site1 dimer (res.50-82)	3NR7	16,232	6×6×6	150	20	470
12	Site1 dimer (res.50-82)	3NR7	16,232	6×6×6	500	20	470
13	Site1 dimer (res.50-82)	3NR7	16,232	6×6×6	150	42	470
	Total						5920

[a] Sequence of S. typhimurium is shown in Fig. 1.

Figure S5: Overview of PMF procedures.

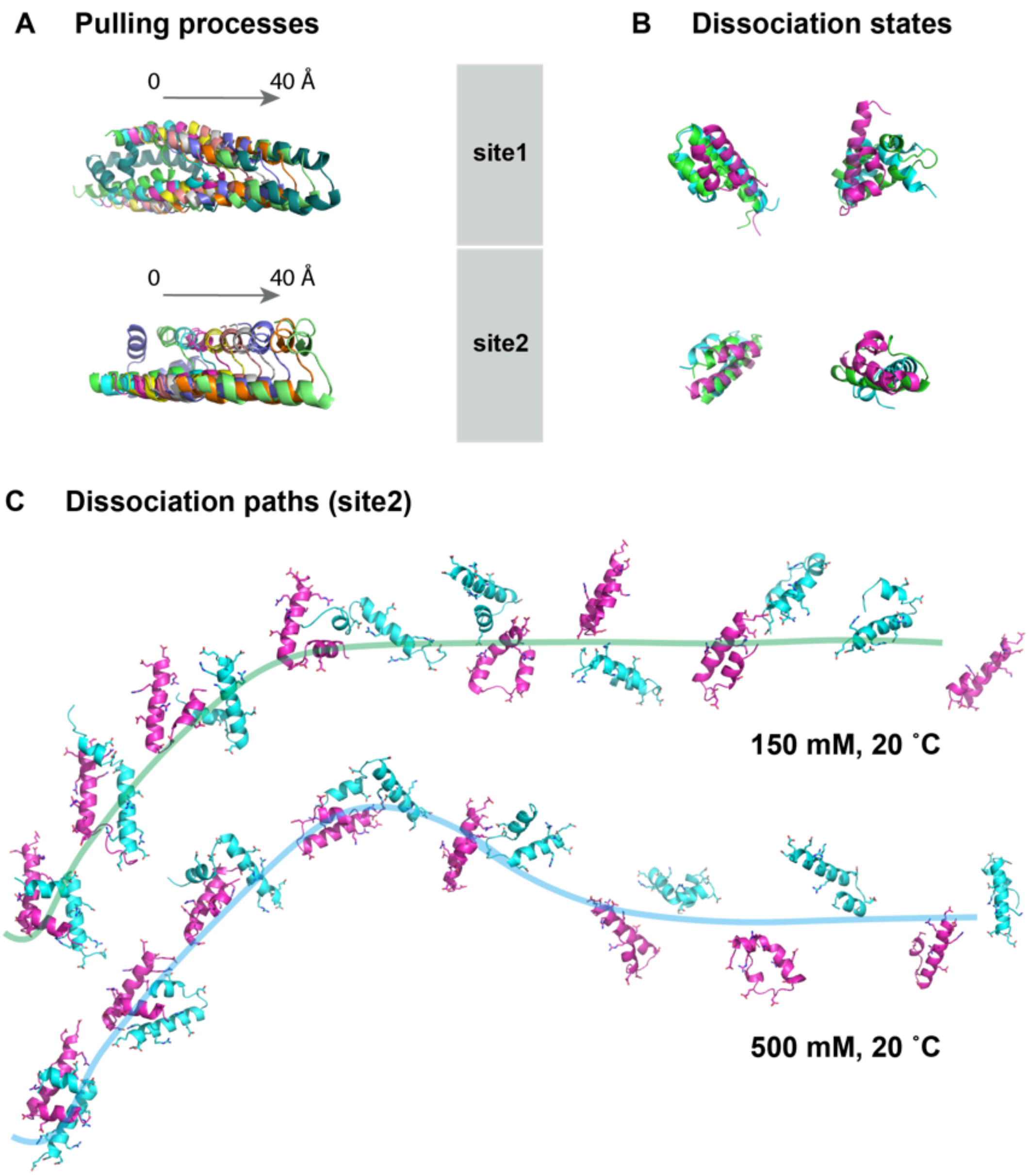

(**A**) Pulling processes of dimeric site1 and site2 presented by 9~10 replicas ("windows"), along which the separation distance range of two monomers gradually increase by 4~5 Å in the span of 0~40 Å in preparation for PMF calculations. In the pulling procedure, we fixed the backbone position of one monomer (left) while pulling the backbone of the other monomer (right) in the arrow direction with a force constant of 16 kcal/mol·A^2 and a pulling speed of 2.5 Å/ps under NVT ensemble. The pulling processes give us a basic idea that it is appropriate to choose the distance between the backbone COM of two monomers as the collective variable. (**B**) Dissociation states of site1 and site2 under three simulation conditions. The initial and final states of a monomer are similar with very little helicity loss. (**C**) The final snapshots of the first eight PMF "windows" of site2 in (i) 150 mM NaCl at 20 ˚C and (ii) 500 mM NaCl at 20 ˚C, which are connected by green and blue curves that reproduce the corresponding PMF plots in Fig. 3E respectively.

Figure S6.

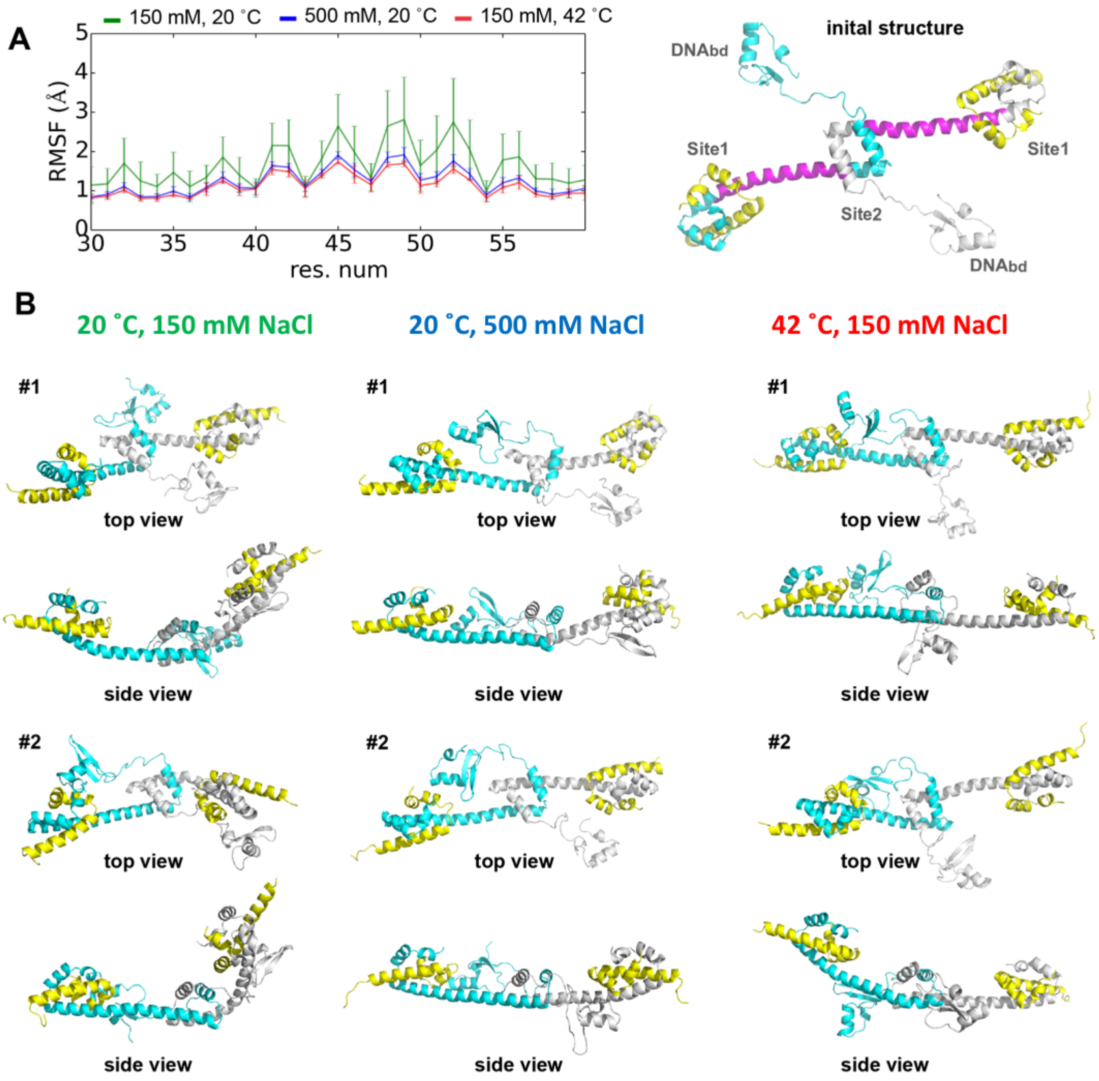

(**A**) Plot of the average residue fluctuation (RMSF) for the middle region (res. 30-60, colored magenta in the initial structure on the right) of H-NS after superpositions on the crystal structure (PDBID: 3NR7). The data were averaged with standard deviations (SD) as error bars from two replica simulations. Larger RMSFs indicate larger bending dynamics within a monomer. (**B**) Finial snapshots of H-NS under regular, high salt, and heat conditions from two replicas (labeled as #1 and #2) each in top and side views. Generally, random bending at the central α3 helix was observed, but it was not correlated with environmental changes in salt or heat.

.

Figure S7.

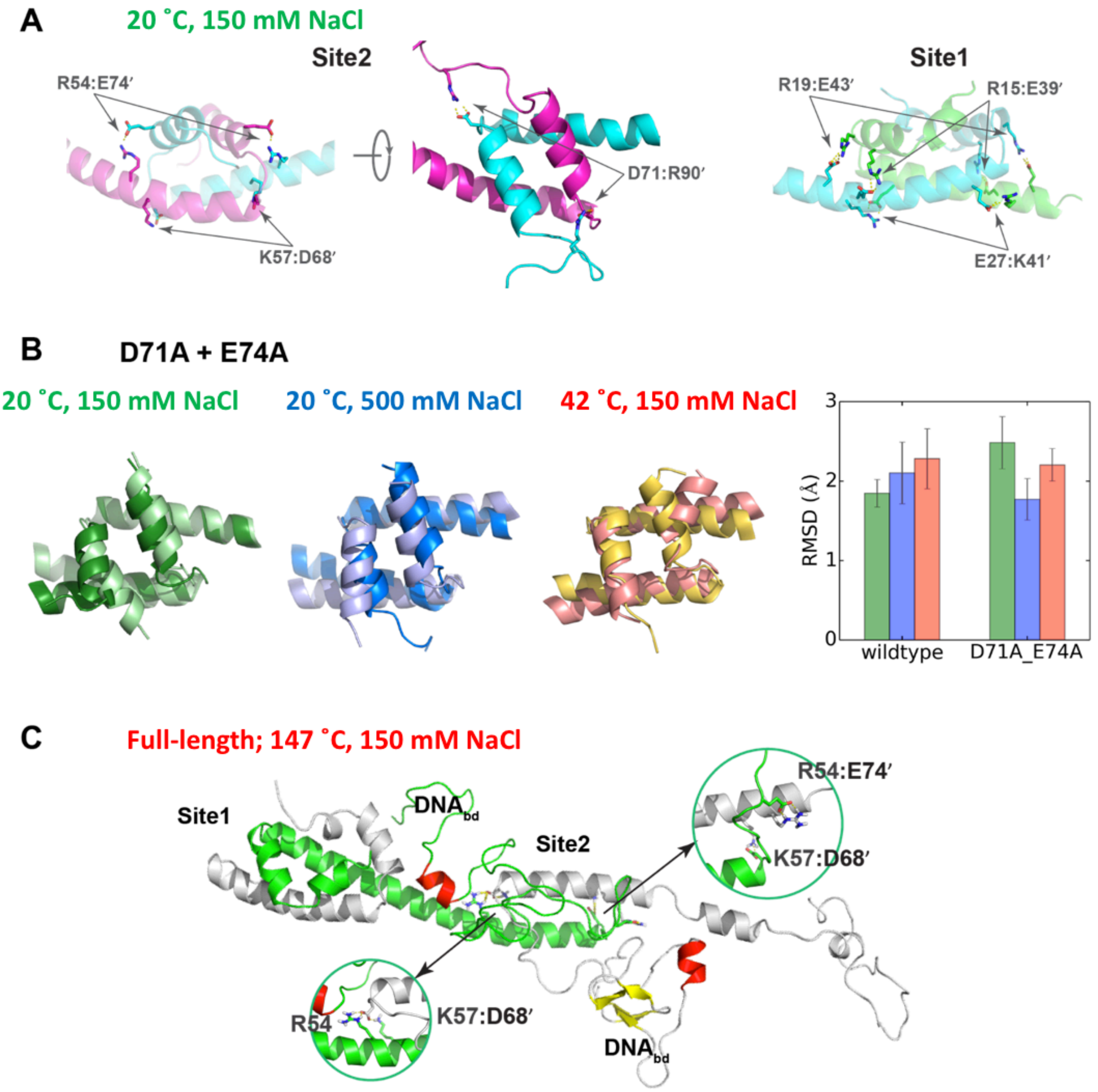

(**A**) Key ionic bonding pairs in site2 and site1 of H-NS respectively illustrated by a final snapshot of 200-ns MD simulations at regular condition (150 mM NaCl at 20 ˚C). (**B**) Stability of site2 with two single point mutations on two key acid residues in α4 region, D71A and E74A. These mutations abandon the natural ionic pairs, R54:E74' and D71:R90', with which H-NS gets the smallest affect from salinity and exhibits relatively large conformational change at regular and high temperature conditions indicated by RMSD measurements. (**C**) Stability of site2 and site1 of wildtype H-NS illustrated by the final snapshot of a 700-ns MD simulation under excessive temperature. Site1 remains in a stable dimerized structure while site2 lost helicity at the α4 region. The R54:E74' pair got disturbed and the helix α4 of site2 turned into coil conformation. Notably, for the middle monomer, no bending was observed at the central α3 helix, largely due to the closely associated DNA binding domain.

Figure S8.

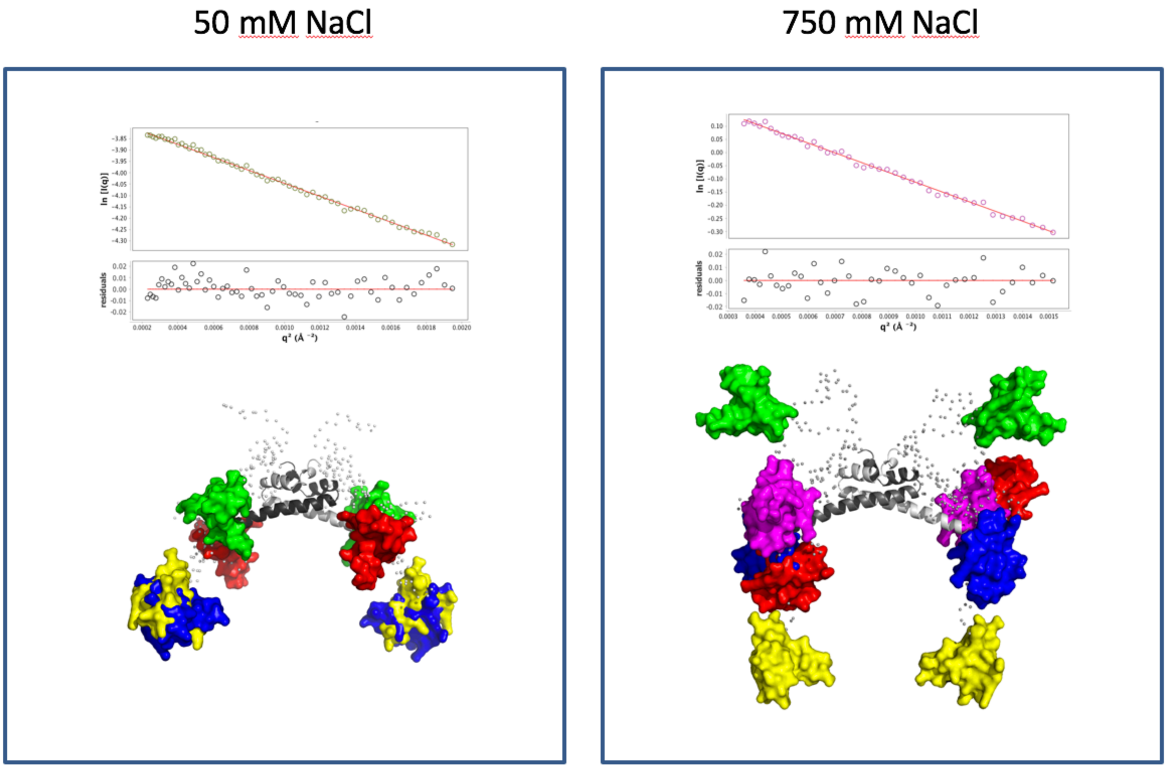

Guiner plot (top) and EOM model (bottom) obtained for SAXS measurements on H-NSΔs2 at 50 mM and 750 mM NaCl. Model representation shows the superimposition of the ensemble established by EOM. Site1 is shown in light and dark grey ribbons. DNAbds are coloured according to each model contributing to the ensemble. Note that the positions obtained do not strictly correspond to experimentally obtained atomic coordinates, but only illustrate the type of positions needed to best recapitulate the data. The information obtained is that even in the low salt condition, there is a dynamic equilibrium between 'closed' and 'open' H-NS conformations. In the high salt condition, the open condition is markedly more prevalent.

Figure S9: Biophysical assessment of interactions between the H-NS N- and C-terminal regions.

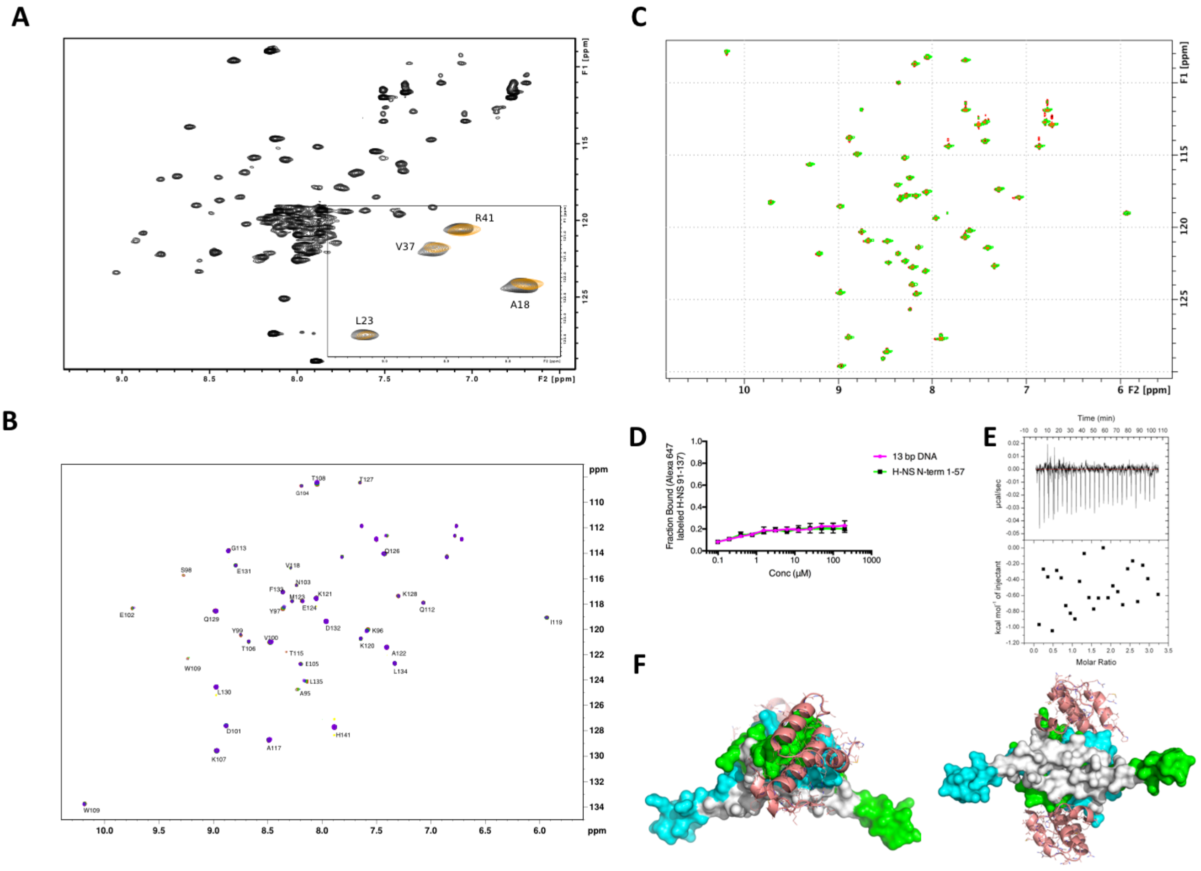

(**A**) ^{1}H-^{15}N HSQC spectra of ^{5}N-labelled H-$NS_{2\text{-}57}$. The inlay shows an example of the effect (chemical shift moving and broadening) of titrating H-$NS_{84\text{-}137}$. Black: apo H-$NS_{2\text{-}57}$; orange: H-$NS_{2\text{-}57}$: H-$NS_{84\text{-}137}$ at a ratio of 1: 2. (**B**) Overlay of ^{1}H-^{15}N HSQC spectra, where unlabelled H-$NS_{2\text{-}57}$ was added to ^{15}N-labelled H-$NS_{84\text{-}137}$ (magenta) until the ratio of 0.5:1 (blue), 1:1 (green) and 2:1(red). (**C**) Overlay of 1H-15N HSQC spectra, 15N-labeled H-$NS_{91\text{-}137}$ (red) with addition of unlabelled H-$NS_{2\text{-}57}$ until the ratio of 2:1 (green). (**D**)Interactions between H-$NS_{91\text{-}137}$ A-T rich 13bp DNA and H-$NS_{2\text{-}57}$ were assessed using MST. Data in (**C**) and (**D**) are mean ± S.D, n = 3. (**E**) ITC measurement. 200 µl of H-$NS_{2\text{-}57}$ was placed in the measurement cell at a concentration of 20 µM. H-$NS_{91\text{-}137}$ was kept at a concentration of 300 µM in the injection syringe. Titrations were performed at 25 °C with an initial injection of 0.4 µl, followed by 25 injections of 2 µl. (**F**) Hha (light-red ribbon) binds to H-NS on sites that are close to, and partly overlapping with, the interaction site for the H-NS C-terminal region (white surface). The structure of the HhA:H-NS complex is taken from PDB 4icg.
